# Development and validation of a standardized emotional music database based on multidimensional affective ratings

**DOI:** 10.3389/fpsyg.2025.1695114

**Published:** 2025-10-22

**Authors:** Rentana Wu, Yuru Huang, Xinhong Jin

**Affiliations:** ^1^School of Arts, Shanghai University of Sport, Shanghai, China; ^2^School of Psychology, Shanghai University of Sport, Shanghai, China; ^3^Center for Exercise and Brain Science, Shanghai University of Sport, Shanghai, China; ^4^Key Laboratory of Motor Cognitive Assessment and Regulation, Shanghai University of Sport, Shanghai, China

**Keywords:** emotional music database, affective ratings, valence-arousal model, music therapy, cluster analysis

## Abstract

**Introduction:**

Music is an effective medium for eliciting and regulating emotions and has been increasingly applied in therapeutic contexts. Yet the absence of standardized and validated music stimulus databases limits reproducibility and application in psychological and clinical research. This study aimed to develop a culturally inclusive therapeutic music database and to examine its affective validity and reliability.

**Methods:**

A total of 234 participants rated 87 instrumental excerpts from Chinese and Western traditions, spanning classical, traditional, and popular genres, along six dimensions: valence, arousal, expressiveness, familiarity, liking, and perceived tempo.

**Results:**

Descriptive analyses indicated moderate to high ratings across dimensions, and reliability testing confirmed strong internal consistency across repeated evaluations (test–retest rs = 0.74–0.89, *p*s < 0.001). Correlation analyses demonstrated a coherent internal structure among the six dimensions. Exploratory factor analysis further supported a unidimensional affective–perceptual factor (KMO = 0.75, *p* < 0.001), explaining 79.2% of the variance. Cluster analysis yielded three distinct categories: Positive–Energizing (*n* = 27), Neutral–Relaxing (*n* = 19), and Negative–Reflective (*n* = 14), which aligned significantly with expert-defined classifications [χ^2^(4) = 55.9, *p* < 0.001, Cramér’s V = 0.57].

**Discussion:**

Based on these results, a final set of 60 validated excerpts was retained to form a standardized therapeutic music library. This resource offers a multidimensional, cross-culturally grounded, and empirically validated tool to advance emotion research, support cross-cultural comparisons, and guide the design of evidence-based music interventions in psychological and clinical practice.

## 1 Introduction

From calming a restless infant to helping athletes maintain focus or providing comfort during emotional distress, music plays a powerful role in daily emotional life. People often turn to music consciously or unconsciously to regulate mood, relieve stress, or achieve emotional release. These everyday experiences align with a growing scientific recognition of music as an effective tool for emotion induc tion and regulation (Chanda and Levitin, 2013; Koelsch, 2014). As a non-invasive and culturally universal stimulus, music has been increasingly applied in therapeutic contexts, including stress reduction, mood enhancement, and emotional regulation (Hunter et al., 2008; Koelsch, 2011; Zhang et al., 2022). Meta-analytic evidence further confirms that music can modulate both psychological states and physiological markers of stress, such as heart rate, blood pressure, and anxiety levels (de Witte et al., 2020).

Despite its ubiquity and effectiveness, research and clinical applications of music still lack a foundational resource: standardized, validated musical stimulus sets. Current practices often rely on participant-selected or expert-curated excerpts, which are neither fully transparent nor reproducible (Arbinaga et al., 2020; Elliott et al., 2014; Kim et al., 2018; Pettit and Karageorghis, 2020). These practices lack transparency, standardization, and empirical validation, undermining reproducibility and generalizability. Moreover, existing databases are generally limited in scope, often focusing on Western classical music, and do not systematically incorporate multidimensional affective properties. Few consider cultural diversity, genre variation, or multidimensional emotional properties, limiting their utility in applied intervention contexts. This gap highlights the need for a standardized, culturally inclusive music database that can support both research reproducibility and therapeutic application across diverse populations. In contrast, standardized visual stimuli, such as the International Affective Picture System (IAPS; Bradley and Lang, 2007) or Ekman’s facial expression sets (Ekman, 1993), have greatly enhanced reproducibility in emotion research, highlighting the need for comparable resources in music-based studies. Crucially, music’s emotional impact remains relatively stable across demographic variations, such as age, education level, or language background, which often confound visual or textual stimuli. However, the development of such resources remains limited, particularly in non-Western contexts or in settings with specific intervention-oriented goals.

Compared to visual or linguistic stimuli, music offers unique advantages as an emotional stimulus. It is nonverbal, culturally transcendent, and capable of eliciting both positive and negative affective states with intensity and consistency (Aljanaki et al., 2017; Arazi et al., 2015). The circumplex model of affect (Russell, 1980; Russell and Barrett, 1999) has provided a fundamental framework for classifying emotional responses to music, using the two core dimensions of valence (positive–negative) and arousal (calm–activated). While these dimensions capture broad affective qualities, additional perceptual and evaluative dimensions, such as expressiveness, familiarity, liking, and perceived tempo, are necessary to account for the complexity of musical emotion (Juslin and Sloboda, 2010; Scherer, 2004; Zentner et al., 2008). The distinction between the emotion expressed by music and the emotion perceived or felt by the listener is particularly relevant for both psychological research and therapeutic applications (Gabrielsson, 2002).

To address these limitations, the present study aimed to develop a standardized, culturally inclusive, and therapeutically relevant emotional music database. We selected 87 instrumental excerpts from both Chinese and Western traditions, covering classical, traditional, and popular genres. A large sample of university students rated each excerpt on six dimensions: valence, arousal, expressiveness, familiarity, liking, and perceived tempo. This multidimensional framework allows for a comprehensive understanding of how different musical features contribute to emotional impact and supports reproducible classification of music according to affective properties. By integrating multiple musical traditions and multidimensional affective ratings, this framework enables both cross-cultural comparisons and practical application in diverse therapeutic contexts, such as stress reduction, mood modulation, and emotional regulation interventions.

Specifically, the study had three primary objectives: (a) to construct a standardized and validated music database for psychological and therapeutic research, (b) to examine the reliability of multidimensional affective ratings, and (c) to identify distinct clusters of therapeutic music based on empirical data. We hypothesized that (1) valence and arousal would serve as the primary dimensions differentiating categories, (2) secondary dimensions such as expressiveness, familiarity, liking, and tempo would contribute consistent variance to classification, and (3) cluster analysis would yield three meaningful groups corresponding to Positive–Energizing, Neutral–Relaxing, and Negative–Reflective categories. Ultimately, this study aims to provide a reusable, scientifically grounded resource that advances cross-cultural emotion research, informs evidence-based music interventions, and supports the development of sensitive therapeutic practices.

## 2 Materials and methods

### 2.1 Participants

A total of 234 students (aged 18–25) were recruited through online advertisements and university forums. Participants were recruited via both online platform (NaoDao, https://www.naodao.com/) and offline posters on campus. Stratified randomization was used to divide participants into two groups to reduce fatigue effects during music rating. First, the sample was stratified by gender, and within each gender subgroup, participants were randomly ordered using computer-generated random numbers. The first 117 participants (59 males and 58 females) were assigned to Group 1, and the remaining 117 participants (62 males and 55 females) were assigned to Group 2. All participants reported normal hearing and no history of neurological or psychiatric disorders.

Group 1 completed ratings of the first set of music excerpts, and Group 2 rated the second set. In Group 1, data from 117 participants were aged between 18 and 25 years (M = 21, SD = 1). The valid sample for Group 2 included 117 participants aged 20 ± 1.58 years old. All participants were enrolled in a range of academic disciplines, including the humanities, social sciences, natural sciences, and engineering. Prior to participation, all individuals were informed of the study’s purpose and procedures and provided written informed consent. Upon completion of the study, participants received monetary compensation for their time (Table 1).

**Table 1:** Demographic characteristics of participants in Group 1 and Group 2.

Demographic	Group 1 (*n* = 117)	Group 2 (*n* = 117)
Age, mean (±SD) years	21.0 (±1.0)	20.0 (±1.6)
Gender (Male/Female)	59/58	62/55
Undergraduate Education	94	104
Master’s Education	22	12
Doctoral Education	1	1

Values are presented as mean (± SD) for continuous variables and as counts for categorical variables. Group 1 (*n* = 117) and Group 2 (*n* = 117) did not differ significantly in age distribution, gender ratio, or educational background.

### 2.2 Music stimuli and selection

A total of 87 instrumental music excerpts (approximately 60 s each) were compiled through a multi-stage selection process designed to ensure emotional specificity, therapeutic relevance, and acoustic consistency. Candidate tracks were collected from a wide range of sources, including professional music platforms, public music websites, online forums, and peer recommendations. The selection was guided by the goal of accurately representing three affective categories: positive, neutral, and negative in alignment with therapeutic emotional targets.

In the initial selection stage, five domain experts, including professional musicians and specialists in music therapy and music cognition, gathered the music excerpts from multi-platforms using search terms for each category such as positive: “motivational or uplifting music (forte/energico/vivid allegro/brillante/giocoso/con brio dances/marches).” neutral: “soothing or stress-relief music (cantabile/legato/dolce adagio/andante/grazioso piano/instrumental music),” negative: “melancholy or sentimental music (sotto voce/morendo largo/grave/lento nocturne/funeral march).” Finally, the tracks were further applied throughout musical analysis in music structural and expression features, specifically including tempo, melody, tonality, structure/form, dynamics, rhythm, instrumentation, and emotional salience (Martin-Saavedra et al., 2018). Those not meeting inclusion criteria were excluded.

Experimental music tracks editing was conducted using a professional music editing software—Audacity. All music excerpts were converted into WAV format and standardized for playback quality. Specifically, audio parameters were uniformly adjusted to a sampling rate of 8,000 Hz, 24-bit resolution, and stereo sound to ensure consistency across playback systems and research environments. Track durations were normalized to range between 55 and 65 s. Volume levels of each track were standardized across participants prior to the experiment to ensure consistent auditory perception.

The initial music database consisted of 87 music excerpts selected to represent a diverse range of genres, including Western and Chinese music, categorized as Classical and Popular music (see more details in Table 2). These categories were chosen to provide a broad spectrum of cultural and stylistic diversity, which could potentially influence listeners’ emotional responses.

**Table 2:** Distribution of the initial music materials by genre and cultural origin.

Phase	Category	Sub-category	Numbers of Pieces (*n*)	Percentages (%)
Initial Music Database	Western	Classical	8	9.19
		Popular	38	43.68
	Chinese	Classical	7	8.05
		Popular	34	39.08
	Total		87	100
Validated Music Database	Western	Classical	8	13.33
		Popular	24	40
	Chinese	Classical	7	11.67
		Popular	21	35
	Total		60	100

The table shows the number and percentage of Western and Chinese music subcategories (classical and popular) in the initial and validated music databases.

Each music excerpt was classified into one of three affective categories based on experts’ analysis: Positive (*n* = 32): high-valence, high-arousal music; Neutral (*n* = 34): high-valence, low-arousal, and calm-inducing music; Negative (*n* = 21): low-valence, low-arousal, and introspective or melancholic music (Russell, 1980). To minimize familiarity bias while preserving emotional recognition, excerpts were selected to ensure moderate exposure levels within the target population. This strategy was informed by prior research suggesting a curvilinear (inverted U-shaped) relationship between music familiarity and preference (Schellenberg, 2008).

For experimental evaluation, the final pool was randomly divided into two stimulus sets of comparable affective compositions. To assess intra-rater reliability and participant attentiveness, six music excerpts (two from each affective category) were randomly duplicated within each set, resulting in a total of 50 music excerpts per participant.

### 2.3 Rating procedure

Each participant listened to 50 music excerpts presented in a randomized order, either in a quiet laboratory setting using Psychopy (version 2021.2.3) or through an online Qualtrics interface under self-guided but standardized conditions. After listening to each excerpt, participants were instructed to evaluate the music on six perceptual and emotional dimensions using 9-point Likert scales: valence, arousal, expressiveness, familiarity, liking, and perceived tempo. These dimensions were selected to capture both the affective properties of the stimuli and inter-individual variability in perceptual and preferential responses.

Valence referred to the degree of pleasantness the music elicited, with 1 indicating very unpleasant and 9 indicating very pleasant. Arousal measured the level of stimulation or energy induced by the music, ranging from 1 (very calming) to 9 (highly exciting). Expressiveness was defined as the emotional clarity and intensity conveyed by the music, rated from 1 (emotionally flat) to 9 (highly expressive). Familiarity assessed how well the participant felt they recognized or had previously encountered the excerpt, ranging from 1 (completely unfamiliar) to 9 (highly familiar). Liking captured the participant’s degree of personal preference for the music, from 1 (strongly dislike) to 9 (strongly like). Finally, perceived tempo measured the subjective pace of the music, with 1 representing very slow and 9 representing very fast. All rating definitions and anchor points were explained in detail prior to the experiment to ensure a consistent understanding across participants.

To evaluate intra-rater reliability and attentional consistency, approximately 10% of the excerpts (5 out of 50) were randomly duplicated and embedded within each participant’s session. These repeated items were not identified as such, allowing for an unobtrusive assessment of rating stability. Participants were instructed to rely on their immediate impressions and to avoid overthinking their responses in order to capture naturalistic emotional judgments.

### 2.4 Experimental procedure

The experimental procedure was identical across the two groups, with each session lasting approximately 60 min. A total of 234 participants (117 in each group) completed the experiment either in a controlled laboratory environment or via a secure online platform. In both conditions, participants used personal computers with headphones and performed the task individually without any interaction with others. For the online condition, real-time video monitoring was implemented to ensure compliance with the instructions and to maintain participants’ attentional state throughout the session. Stimuli were presented using Psychopy (version 2021.2.3), which was deployed both online and offline to ensure standardized delivery.

All 87 music excerpts were presented in randomized order to minimize order effects. Before the formal session began, participants were provided with detailed instructions and three practice trials, one for each affective category (positive, neutral, and negative), to familiarize themselves with the task interface and rating procedure.

During the main experiment, participants were instructed to listen attentively to each excerpt and then rate it along six dimensions using the number keys (1–9) on the keyboard. The six rating scales (valence, arousal, expressiveness, familiarity, liking, and perceived tempo) were presented one at a time in a fixed sequence immediately after the music playback. Once a rating was submitted, the next dimension appeared automatically. Participants were encouraged to respond based on their immediate impressions and to avoid overthinking.

To minimize fatigue and maintain concentration, participants were prompted to take a short self-paced break after every 10 excerpts. The length of each break was self-paced and determined by the participant. The experimental interface allowed full self-control over rating speed while maintaining consistency in stimulus presentation.

### 2.5 Statistical analysis

All statistical analyses were performed using SPSS 26.0. Descriptive statistics (mean and standard deviation) were calculated for each of the six rating dimensions. To assess the internal consistency and reliability of participant responses, Pearson correlations were computed for the repeated music excerpts within each group. To explore the underlying structure of emotional evaluations, exploratory factor analysis (EFA) was conducted on the full set of ratings.

Given the strong intercorrelations among valence, arousal, and expressiveness, K-means clustering was applied to categorize the 87 music excerpts into three affective groups. A chi-square test was used to evaluate the correspondence between cluster assignments and expert-defined categories. Finally, to evaluate whether the three data-driven music clusters significantly differed in their affective profiles, a multivariate analysis of variance (MANOVA) was conducted on the six rating dimensions. Significant effects were followed up with Bonferroni-corrected post-hoc tests to identify specific dimensions contributing to the cluster differentiation.

## 3 Results

### 3.1 Descriptive results

**Figure 1:**
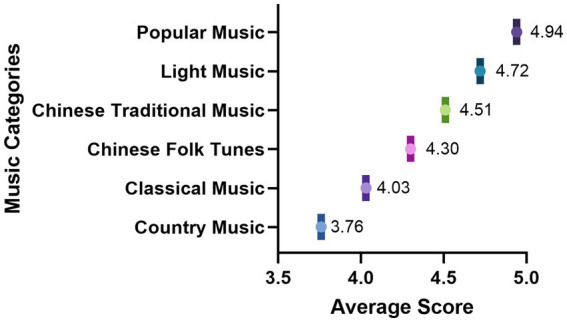
Average preference ratings across music genres. Mean preference ratings are shown for musical genres based on evaluations from participants. Bars represent average ratings. The results highlight genre-related differences in participants’ evaluative responses, providing an overview of general listening preferences across musical traditions.

Descriptive statistics were calculated for all six rating dimensions across the 87 music excerpts, based on the combined data from both participant groups. The results indicated the following means and standard deviations: valence (M = 5.68, SD = 1.77), arousal (M = 5.21, SD = 1.87), expressiveness (M = 5.59, SD = 1.87), familiarity (M = 4.63, SD = 2.39), liking (M = 5.60, SD = 1.94), and perceived tempo (M = 4.79, SD = 1.88). These values suggest that, overall, the musical excerpts elicited moderately high levels of positive affect and emotional expressiveness, with some variability in familiarity and perceived tempo, reflecting a diverse and well-balanced stimulus set suitable for further classification and therapeutic applications (Figure 1).

In addition, no significant gender differences were found in any of the six rating dimensions. Independent samples *t*-tests revealed that male and female participants rated the 87 musical excerpts similarly in terms of valence, arousal, expressiveness, familiarity, liking, and perceived tempo (*ps* > 0.05 for all comparisons). These results suggest a high degree of consistency across genders in the emotional and perceptual evaluation of the musical stimuli.

### 3.2 Rating consistency

To assess the reliability of participants’ evaluations, Pearson correlation coefficients were calculated for 12 repeated music excerpts across the six rating dimensions. The test–retest correlations were high and statistically significant for all dimensions: valence (r = 0.806, *p* < 0.001), arousal (r = 0.888, *p* < 0.001), expressiveness (r = 0.744, *p* < 0.001), familiarity (r = 0.843, *p* < 0.001), liking (r = 0.782, *p* < 0.001), and perceived tempo (r = 0.812, *p* < 0.001). These results indicate acceptable internal consistency and confirm the reliability of participants’ ratings across sessions.

### 3.3 Inter-dimensional correlations

To examine the relationships among the six evaluative dimensions, Pearson correlation coefficients were calculated based on the mean ratings of each musical excerpt (*N* = 87). As shown in Table 3, all dimensions were significantly and positively correlated (*p*s < 0.001), suggesting a coherent internal structure among the affective and perceptual components.

**Table 3:** Inter-dimensional Pearson correlations among the six affective and perceptual rating dimensions.

Dimensions	Valence	Arousal	Expressiveness	Familiarity	Liking	Tempo
Valence	1	0.901^**^	0.976^**^	0.518^**^	0.866^**^	0.793^**^
Arousal		1	0.940^**^	0.528^**^	0.733^**^	0.955^**^
Expressiveness			1	0.442^**^	0.778^**^	0.860^**^
Familiarity				1	0.784^**^	0.442^**^
Liking					1	0.580^**^
Tempo						1

Correlation coefficients are shown for valence, arousal, expressiveness, familiarity, liking, and perceived tempo across all 87 music excerpts. All correlations are significant at *p* < 0.001.

Valence demonstrated strong correlations with expressiveness (r = 0.976), arousal (r = 0.901), liking (r = 0.866), and tempo (r = 0.793), indicating that more pleasant music was also perceived as more emotionally expressive, arousing, and fast-paced, and was more liked overall. Arousal was highly correlated with expressiveness (r = 0.940) and tempo (r = 0.955), consistent with the idea that faster music tends to elicit higher arousal. Familiarity showed moderate correlations with valence (r = 0.518), arousal (r = 0.528), and liking (r = 0.784), suggesting that known music may evoke more favorable emotional responses. These results support the interrelated nature of emotional and perceptual responses to music and provide a sound basis for subsequent factor and cluster analyses.

### 3.4 Factor analysis of affective ratings

To explore the underlying structure of the six affective and perceptual rating dimensions, an exploratory factor analysis (EFA) was conducted using principal component extraction and Varimax rotation. The Kaiser–Meyer–Olkin (KMO) measure verified the sampling adequacy with a value of 0.747, and Bartlett’s test of sphericity was significant (*p* < 0.001), confirming the suitability of the data for factor analysis.

The analysis yielded a single dominant factor with an eigenvalue greater than 1, which accounted for 79.21% of the total variance. This result suggests a highly cohesive evaluative structure, in which all six dimensions (valence, arousal, expressiveness, familiarity, liking, and perceived tempo) contributed meaningfully to a unidimensional affective-perceptual factor. This finding supports the psychological coherence of the rating scales and provides a theoretical basis for subsequent classification and therapeutic categorization of the musical stimuli.

### 3.5 Cluster analysis

Given the high intercorrelations among the six rated dimensions, the selected variables were z-standardized prior to clustering in order to eliminate scale differences and reduce multicollinearity. To further explore the emotional structure of the music database, a K-means cluster analysis was conducted using three core affective rating dimensions: valence, arousal, and expressiveness.

Specifically, the first cluster (*n* = 27) was characterized by high levels of valence, arousal, and expressiveness, corresponding to a Positive profile. The second cluster (*n* = 19) reflected a Relaxing profile, with low arousal, moderate valence, and relatively calm emotional tones. The third cluster (*n* = 14) captured music excerpts with low valence and lower levels of arousal and expressiveness, representing a Negative profile. These data-driven categories closely mirror the initial expert-labeled classifications, which consisted of 32 Positive, 34 Relaxing, and 21 Negative excerpts selected based on theoretical and stylistic criteria.

To assess the alignment between expert categorization and the clustering results, a chi-square test of independence was conducted on the cross-tabulated frequencies. The analysis revealed a significant association between the two classification systems, χ^2^(4) = 55.93, *p* < 0.001, indicating a strong relationship between expert categorizations and participant-informed emotional clustering. Cramér’s V = 0.57, suggesting a large effect size. The results further demonstrate the reliability and psychological coherence of the music database, reinforcing its applicability as a foundational tool for affective music therapy and emotion-oriented intervention programs.

Based on the standardized cluster centers for the six rating dimensions, Cluster 1 exhibited high scores in valence, arousal, and expressiveness, suggesting a profile of emotionally uplifting and stimulating music, labeled as Positive–Energizing. Cluster 2 showed low scores across affective dimensions, indicating emotionally subdued and reflective characteristics, hence labeled as Negative–Reflective. Cluster 3 was marked by moderate valence, lower arousal, and lower expressiveness, aligning with a Neutral–Relaxing emotional profile. The final validated database consisted of 60 music excerpts. These excerpts were re-categorized into Western and Chinese subcategories (Classical and Popular). The distribution of genres in both the initial and validated databases is provided in Table 2, showing the number and percentage of each genre in both datasets.

### 3.6 Differences in affective ratings across clusters

To examine whether the three clusters differed in their affective profiles, a multivariate analysis of variance (MANOVA) was conducted using the six rating dimensions as dependent variables. The results showed a significant multivariate effect of cluster membership (*p*s < 0.001), indicating that the clusters varied systematically across the emotional dimensions. Post-hoc comparisons showed that Cluster 1 (Positive) received the highest ratings in valence, arousal, and liking; Cluster 2 (Negative) had the lowest scores on these dimensions; and Cluster 3 (Relaxing) showed moderate valence but lower arousal, aligning with its calm-inducing profile.

**Figure 2:**
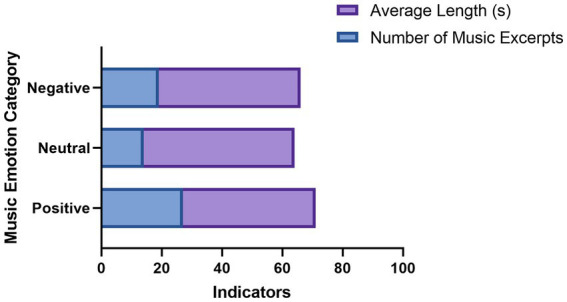
Distribution of music excerpts across emotional categories in the finalized therapeutic music database. The y-axis represents emotional categories (positive, neutral, negative), and the x-axis shows two metrics: the number of excerpts and their average lengths. Bars illustrate differences in both quantity and duration across categories.

These findings support the validity of the clustering solution and suggest that the categorized music excerpts represent distinct affective types relevant to therapeutic applications (Figure 2).

## 4 Discussion

This study aimed to develop a standardized, multidimensional emotional music database that integrates both empirical rigor and therapeutic relevance. By evaluating 87 musical excerpts drawn from diverse cultural traditions and genres, we sought to construct a validated resource for emotion research and intervention. Six core subjective dimensions, valence, arousal, expressiveness, familiarity, liking, and perceived tempo, were systematically assessed to capture the affective and perceptual characteristics of each musical piece. The results revealed consistently moderate to high ratings on affective dimensions, with no significant gender differences, and demonstrated high test–retest reliability across repeated excerpts. Moreover, strong intercorrelations were observed among the six dimensions, and exploratory factor analysis yielded a single latent factor that accounted for over 79% of the variance, suggesting a cohesive underlying affective-perceptual structure. Subsequent cluster analyses further identified three distinct emotional profiles, positive, neutral and negative that closely aligned with expert categorizations, thereby reinforcing the validity and applicability of the database.

The descriptive results indicate that the selected excerpts elicited moderately high levels of valence, arousal, and expressiveness across participants, with acceptable variation in familiarity and tempo. This balance suggests that the musical set is both emotionally engaging and sufficiently diverse, fulfilling its role as a flexible resource for affective research and therapeutic use. Notably, no significant gender differences were found in the ratings across all six dimensions, underscoring the consistency and generalizability of the emotional responses elicited by the stimuli. The reliability of participants’ evaluations was further confirmed through high test–retest correlations across all dimensions, with particularly strong stability observed for core affective attributes such as valence and arousal. Together, these findings highlight the robustness of the evaluation procedure and provide critical evidence for the reproducibility and standardization of the database, ensuring its utility across different participant groups and experimental contexts.

Correlational analyses among the six rating dimensions revealed a coherent and tightly interconnected evaluative structure. Valence was strongly associated with expressiveness, arousal, liking, and tempo, suggesting that positively valenced music tends to be perceived as more emotionally expressive, arousing, faster-paced, and subjectively preferred. Arousal was likewise highly correlated with expressiveness and tempo, reinforcing the notion that tempo is a critical perceptual cue for emotional activation in music (Juslin and Västfjäll, 2008). Familiarity showed moderate correlations with core affective dimensions, suggesting that known music may enhance emotional resonance, possibly through mechanisms of memory and autobiographical relevance. These strong inter-dimensional relationships offer empirical support for the integrative nature of emotional and perceptual music processing.

The exploratory factor analysis provided further insight into the underlying structure of these subjective ratings. A single latent factor explained over 79% of the variance, indicating that the six dimensions may reflect a unified evaluative construct. This finding aligns with prior theories suggesting that listeners tend to form holistic emotional impressions of music, rather than isolating discrete perceptual or affective elements (Gabrielsson, 2002). The convergence of affective and aesthetic judgments within a single factor has important implications for the design of music-based interventions, as it suggests that therapeutic effects may be mediated through integrated emotional-perceptual pathways.

To classify the emotional characteristics of the musical excerpts, a K-means clustering analysis was conducted using three key dimensions. The analysis yielded three distinct clusters, which were subsequently interpreted as positive, neutral, and negative profiles. Cluster 1 was characterized by high levels of positivity, activation, and emotional expressivity, often associated with mood enhancement and motivational engagement. Cluster 2 represented music that was emotionally subdued, with low arousal and moderate valence, aligning with calm-inducing or contemplative listening contexts. Cluster 3 included music with low valence and low expressiveness, matching profiles typically linked to emotional introspection or sadness. The validity of this data-driven classification was tested against expert-defined emotional categories. A chi-square analysis revealed a significant and strong association between the participant-derived clusters and the *a priori* expert labels, confirming that subjective emotional appraisals align closely with professional intuition. This convergence enhances confidence in the classification scheme and demonstrates the value of combining empirical participant data with expert curation to ensure both psychological relevance and clinical validity.

Further analyses confirmed that the three clusters differed significantly across all six rating dimensions. Post-hoc comparisons revealed clear affective distinctions. Cluster 1 received the highest ratings in valence, arousal, and liking; Cluster 2 scored moderately on valence and low on arousal and expressiveness; and Cluster 3 exhibited the lowest ratings across most dimensions. These results not only validate the clustering solution but also provide affective “fingerprints” for different music types, enabling targeted applications in emotion regulation.

Although the primary classification in this study was based on affective ratings rather than genre, we recognize that genre can influence listeners’ emotional responses, particularly through cultural familiarity and stylistic expectations. To ensure transparency, we report the genre distributions of both the initial 87 music excerpts and the final 60 validated excerpts (see Table 2). Importantly, the affective validity of the database was maintained across both Western and Chinese music subcategories, confirming that emotional responses were consistent across different musical traditions. Future research could explore how genre may act as a moderating factor in emotional responses.

Theoretical implications of these findings are multifold. First, they extend support for the dimensional model of emotion (Russell, 1980), highlighting valence and arousal as core affective axes in music perception. However, the inclusion of expressiveness as a third dimension proved particularly valuable, allowing for differentiation between perceived emotional intent (what the music expresses) and felt emotion (what the listener experiences), a distinction critical in therapeutic settings (Hunter et al., 2008). Second, the high internal coherence of the database suggests that musical emotion processing may operate as a gestalt-like appraisal system, where discrete dimensions inform a unified emotional impression.

Practically, the validated emotional clusters provide a robust foundation for constructing emotion-specific music playlists. Positive music may be particularly effective in interventions targeting depression, fatigue, or apathy by stimulating affective and motivational systems. Neutral pieces may be suited for anxiety reduction, mindfulness training, or sleep facilitation due to their calming and non-intrusive character. Negative music, though less commonly used in clinical practice, may facilitate emotional catharsis or processing in grief or trauma-related interventions. These categorized excerpts, validated across diverse listeners and cultures, form the core of a reusable therapeutic music library with translational potential in clinical psychology, psychiatry, and well-being science.

Despite its strengths, the study has several limitations. The participant sample, primarily young adults aged 18–25, may limit the generalizability of the findings to broader or clinical populations. Future research should include more diverse age groups and clinical samples to explore potential differences in emotional responses across the lifespan. While the current database spans Western and Chinese music, it lacks representation of other cultural genres, such as African and Middle Eastern music. Expanding the music selection to include a broader variety of genres and traditional forms would improve the cross-cultural relevance of the database. Moreover, the music selection, though guided by expert judgment, carries inherent subjectivity. Future studies might consider the role of familiarity with specific pieces, as emotional responses can be influenced by prior exposure and personal associations. Finally, this study did not account for the potential impact of sociocultural factors or participants’ emotional states. Incorporating these factors could offer a more comprehensive understanding of how music is emotionally processed.

In conclusion, this study offers a validated, culturally inclusive, and emotionally informative music stimulus database grounded in both empirical evaluation and expert classification. The findings highlight the integrated nature of emotional music perception and provide a theoretically coherent and practically useful foundation for future research and intervention. By bridging scientific precision with therapeutic relevance, this music library advances the field toward more standardized and effective applications of music in emotion science and mental health care.

## Data Availability

Data supporting the findings of this study are available from the corresponding author upon reasonable request.
